# Supplementary material for: Molten Sn solvent expands liquid metal catalysis
Source: Nat Commun. 2025 Jan 21;16:907. doi: 10.1038/s41467-025-56222-0 (PMC11751482; doi:10.1038/s41467-025-56222-0)
Supplement: Supplementary file 1 — Supplementary Information [file 41467_2025_56222_MOESM1_ESM.pdf]

Supplementary Information for

Molten Sn solvent expands liquid metal catalysis

Junma Tang[1,2,3]*, Nastaran Meftahi[4], Andrew J. Christofferson[5,6], Jing Sun[7], Ruohan Yu[2], Md. Arifur Rahim[1,8], Jianbo Tang[2,9], Guangzhao Mao[2,10], Torben Daeneke[11], Richard B. Kaner[12,13], Salvy P. Russo[5,6], Kourosh Kalantar-Zadeh[1,2]*

[1]School of Chemical and Biomolecular Engineering, The University of Sydney, Sydney 2006, New South Wales, Australia

[2]School of Chemical Engineering, University of New South Wales (UNSW), Sydney 2052, New South Wales, Australia

[3]School of Chemistry, Xi'an Jiaotong University, Xi'an 710049, China

[4]Department of Civil and Construction Engineering, Swinburne University of Technology, Melbourne, Victoria, Australia.

[5]School of Science, STEM College, RMIT University, Melbourne, Victoria, Australia

[6]ARC Centre of Excellence in Exciton Science, School of Science, RMIT University, Melbourne, Victoria, Australia

[7]Centre for Plasma Biomedicine, School of Electrical Engineering, Xi'an Jiaotong University, Xi'an, Shaanxi 710049, China.

[8]Department of Chemical and Biological Engineering, Monash University, Clayton, VIC 3800, Australia

[9]School of Engineering and Research Center for Industries of the Future, Westlake University, Hangzhou 310030, China

[10]School of Engineering, Institute for Materials and Processes, The University of Edinburgh, Edinburgh EH9 3FB, U.K.

[11]School of Engineering, RMIT University, Melbourne, Victoria, Australia

[12]Department of Chemistry and Biochemistry and California NanoSystems Institute, University of California, Los Angeles, Los Angeles, California 90095, United States

[13]Department of Materials Science and Engineering, University of California, Los Angeles, Los Angeles, California 90095, United States

*Correspondence to: junma.tang@xjtu.edu.cn (J.T.), kourosh.kalantarzadeh@sydney.edu.au (K.K.-Z.)

Supplementary discussions

Discussions regarding the cyclic voltammetry experiments (Supplementary Fig. 1):

Cyclic voltammetry was conducted to investigate atomic distributions on the surface of molten alloys. Molten Sn, $SnCu_{0.0094}$, $SnIn_{0.1034}$ and $SnIn_{0.1034}Cu_{0.0094}$ were prepared and rapidly frozen to serve as the working electrodes. By using Sn as the working electrode, the oxidation peak was observed at around -0.85 V (Supplementary Fig. 1a). Using $SnCu_{0.0094}$ as the working electrode, the peak at -0.85 V was assigned to the oxidation of Sn (Supplementary Fig. 1b). No additional significant oxidation peak was observed, suggesting that Cu atoms remain beneath the interfacial layer within the Sn solvent. The peaks centered at -0.85 V and 1.10 V can be assigned to the oxidation of Sn and In, respectively, when $SnIn_{0.1034}$ was employed as the working electrode (Supplementary Fig. 1c). This observation indicates that In atoms can remain on the interfacial layer within molten Sn. Notably, one additional peak at 0.21 V was observed by using $SnIn_{0.1034}Cu_{0.0094}$ as the working electrode, originating from the oxidation of Cu (Supplementary Fig. 1d). These results suggested that Cu atoms can reach to the interfacial layer in the presence of In atoms on the surface of molten $SnIn_{0.1034}Cu_{0.0094}$ alloy. The cyclic voltammetry experiment outcomes regarding the relative distributions of liquid In and Cu atoms in molten Sn solvent are in accordance with the computational simulations, further confirming our proposed reaction mechanisms.

Calculating the catalytic area of the catalyst:

(1) Using a bulk $SnIn_{0.1034}Cu_{0.0094}$ droplet as the catalyst, its surface was considered as the catalytic area due to its direct interaction with the liquid hydrocarbons. Given the droplet's spherical form at the reaction temperature, the surface area (S_1) can be approximately determined using:

$$S_1 = 4\pi r^2 \quad \text{(S1)}$$

where r presents for the radius of the $SnIn_{0.1034}Cu_{0.0094}$ droplet, and r can be calculated based on the volume of the droplet (V) as:

$$V = \frac{4}{3}\pi r^3 \quad \text{(S2)}$$

The volume of the droplet can be easily determined based on the catalyst's weight (m) in the reaction system and the density (d) of the $SnIn_{0.1034}Cu_{0.0094}$ catalyst. Given the minimal amounts of In and Cu in the $SnIn_{0.1034}Cu_{0.0094}$ catalyst, it is reasonable to approximate the catalyst's density with that of Sn (7.265 g/cm^3). Therefore, when using 5.0 g of bulk $SnIn_{0.1034}Cu_{0.0094}$ as the catalyst, the surface area was calculated to be approximately 3.8 cm^2.

(2) When 0.2 g of $SnIn_{0.1034}Cu_{0.0094}$ particles were loaded on the glass microfiber filter papers as the catalyst, the catalytic area can be estimated to be the surface area of these particles. Given the medium diameter (R) of the particle is ~1200 nm, the volume of each particle can be calculated by using equation (S2). Then, the total count of particles (n) can be derived from the volume of an individual particle and the total volume of the alloy. Therefore, the surface area of $SnIn_{0.1034}Cu_{0.0094}$ particles (S_2) can be calculated based on the following:

$$S_2 = n4\pi(\frac{R}{2})^2 \quad \text{(S3)}$$

Hence, the total surface area of the particles was calculated to be ~1360 cm^2, which can be roughly considered as the catalystic area of the $SnIn_{0.1034}Cu_{0.0094}$ catalyst.

Turnover number (TON) estimation calculation:

TON of a catalytic reaction can be calculated based on the amount of the obtained target product and the catalytic materials (S4, both in molar scales).

$$\text{TON} = \frac{n_{product}}{n_{catalyst}} \tag{S4}$$

For the long-term experiment, ~0.2 g of $SnIn_{0.1034}Cu_{0.0094}$ particles were loaded on the glass microfiber filter papers for a continuous 48-hour reaction. During the reaction, the total amount of produced H_2 was estimated to be ~0.0175 mol. In the reaction system, only the atoms on the surface of liquid alloy were involved in the catalytic reaction. The total surface area of 0.2 g of $SnIn_{0.1034}Cu_{0.0094}$ particles was calculated to be ~1360 cm^2.

Given the atomic size of Sn (covalent radius of ~122 pm), the number of Sn atoms on the surface is estimated to be ~3.34×10^{18} (~5.50×10^{-6} mol). Based on atomic ratio, the number of Cu atoms at the interfacial layer can be calculated to be ~5.17×10^{-8} mol. According to our proposed reaction mechanism, the presence of Cu atoms on the surface is essential for H_2 generation, which can be used for the calculation of TON. Hence, the TON is calculated to be ~3.4×10^5, which indicated the propylene production was a catalytic process.

Calculations and the associated discussions of Arrhenius equation (Supplementary Fig. 9)

Originating from the dynamicity and mobility of liquid atoms, AIMD simulations face limitations for molten alloys. The simulation boxes of liquid metallic atoms are small and periodic, and the timescales are short. Moreover, due to the prohibitive computational cost of modelling liquid hexadecane interfaced with liquid metal, we are only able to model a single hexadecane molecule. For thorough simulations of liquid metal catalysis, larger systems for longer times are required, but it will be an ongoing multi-year project and outside the scope of this current work.

Arrhenius equation was employed in this case to gain comprehensive understandings of the activation energy. We conducted the experiments at different reaction temperatures by using $SnIn_{0.1034}Cu_{0.0094}$ alloy as catalyst and hexadecane as feedstock. As shown in Supplementary Table

4, the efficiency for H_2 production increased at higher reaction temperatures. Based on these experimental results, a plot of ln$k$ *vs.* $1/T$ was obtained based on the following Arrhenius equations (S5 and S6):

$$k = Ae^{\frac{-E_a}{RT}} \quad \text{(S5)}$$

$$\ln k = \frac{-E_a}{RT} + \ln A \quad \text{(S6)}$$

where k represents the rate constant, E_a is the activation energy, R is the gas constant (8.3145 J/K mol), T is the temperature expressed in Kelvin, and A is the Arrhenius factor or frequency factor.

According to the Arrhenius plot presented in Supplementary Fig. 9, $-\frac{E_a}{R}$ equals to -12860 K, and E_a is calculated to be ~1.06×10^5 J/mol. Therefore, the activation energy for a single molecule is estimated to be around 1.10 eV.

Discussions of the distinct atomic structures in Ga and Sn solvent (Supplementary Fig. 10):

In a previous study, decane was selectively converted into propylene at the interface of Ga-based liquid alloy $GaSn_{0.029}Ni_{0.023}$[1]. Here we show that H_2 can be selectively synthesized from hexadecane by using Sn-based liquid medium $SnIn_{0.1034}Cu_{0.0094}$ as the catalyst. Despite the fact that in both cases the first step was the removal of H from C-2 with the C-2 stabilized by a surface Sn atom, the reaction pathways were fundamentally altered owing to the distinct atomic structures. The fluidic structures of metallic atoms in each system dominate the reaction route. In the $GaSn_{0.029}Ni_{0.023}$ system, the surface H is stabilized by two activated Ga above a Ni, which allows the H to arrive at a position where it can interact directly with the C-4 carbon, facilitating the breaking of C-3 and C-4 bonds and resulting in propylene and heptane products. On the other hand, in the $SnIn_{0.1034}Cu_{0.0094}$ system, the H interacts directly with Cu and therefore is more embedded

in the surface in an unfavorable distance and configuration for reaction with the C-4 carbon. Although it requires more energy to break the C-H bond than the C-C bond, the atomic configuration in the $SnIn_{0.1034}Cu_{0.0094}$ system nevertheless favors the production of H_2 rather than propylene or other products.

Comparison with other reported materials (Supplementary Table 10):

Currently, both solid-state catalysts and Ga-based liquid alloys have been used for dehydrogenation reactions of hydrocarbons. As shown in Supplementary Table 10, we mainly compared the reaction conditions and efficiency of our reaction system with other catalysts for dehydrogenation reactions. Additionally, we discussed the costs of catalytic materials.

For solid-state catalysts, precious metals, such as Pt, Pd and Ru, are often involved for the preparation of catalysts. Also, high reaction temperatures, normally over 500 °C, are required to trigger the dehydrogenation reactions. Indeed, some of these catalysts demonstrated high reactivity for hydrocarbon dehydrogenations. However, the costs of these solid catalysts normally range from ~US$25 to ~US$100 per gram (reference: stock price Oct. 2024). The harsh operating conditions and the high cost of the catalytic materials restrict the applications of these catalysts.

A series of Ga-based liquid metal catalysts were also developed for dehydrogenation reactions (Supplementary Table 10). Owing to the relatively high price of Ga, preparing one gram of these Ga-based catalysts require ~US$4 per gram (reference: stock price Oct. 2024). High reaction temperatures are still demanded in these cases. Hence, these Ga-based alloys still face limitations for dehydrogenation reactions.

In this work, molten Sn was used as the solvent, without involving precious metals for catalyst synthesis. Synthesizing one gram of $SnIn_{0.1034}Cu_{0.0094}$ catalyst only costs ~US$0.7 (reference:

stock price Oct. 2024), which is more than >35 times cheaper than the solid-state catalysts and >5 times cheaper than Ga-based alloys presented in Supplementary Table 10. Meanwhile, this Sn-based reaction system can facilitate the dehydrogenation reactions at a lower reaction temperature of 260 ℃.

The efficiency of $SnIn_{0.1034}Cu_{0.0094}$ alloy was further calculated based on the weight of catalytic materials for the direct comparison with other materials. In our case, only the liquid atoms on the surface were involved in the catalytic reaction. Given the surface area of the $SnIn_{0.1034}Cu_{0.0094}$ particles and the amount of produced H_2, the efficiency of this reaction system by using canola oil as feedstock was estimated to be ~9.0×10^{-3} $mol\cdot min^{-1}\cdot g_{catalyst}^{-1}$ (Supplementary Table 10). The efficiency of this Sn-based alloy was comparable to many of the previously reported catalysts. Despite some Pt-based solid catalysts revealed higher efficiency, the high costs of such catalytic materials and harsh operating conditions undermine many of the practical values of these catalysts. In comparison, our approach exhibits a few obvious advantages including use of renewable feedstocks, low operating temperature and low cost of catalysts.

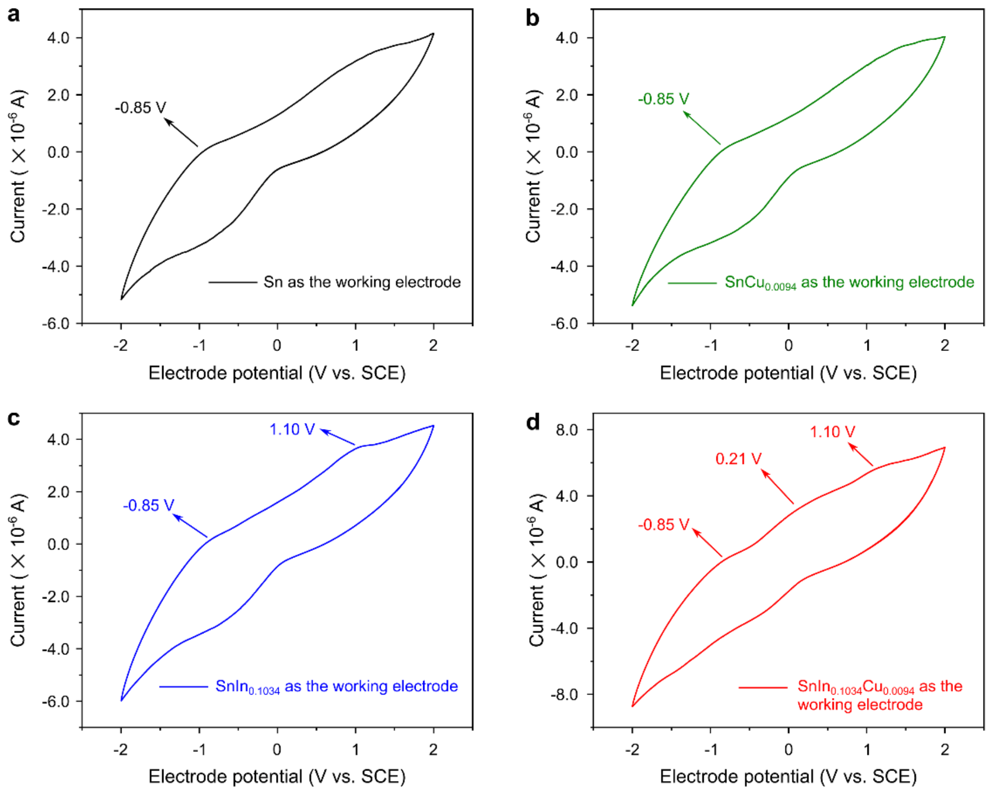

Supplementary Fig. 1 | Cyclic voltammetry experiments by employing different alloys as the working electrode. a-d, Cyclic voltammograms obtained by using Sn (a), $SnCu_{0.0094}$ (b), $SnIn_{0.1034}$ (c) and $SnIn_{0.1034}Cu_{0.0094}$ (d) as the working electrodes. The cyclic voltammetry experiments were conducted to investigate the relative positions of the dispersed atoms in molten $SnIn_{0.1034}Cu_{0.0094}$ alloy with reference to the surface and in comparison to Sn, $SnCu_{0.0094}$, and $SnIn_{0.1034}$. Here, Sn, $SnCu_{0.0094}$, $SnIn_{0.1034}$ and $SnIn_{0.1034}Cu_{0.0094}$ were painted onto an indium tin oxide (ITO) substrate at 260 °C. After quick freeze of the liquid alloys using liquid nitrogen, the solidified Sn, $SnCu_{0.0094}$, $SnIn_{0.1034}$ and $SnIn_{0.1034}Cu_{0.0094}$ on the suberates were used as the working electrodes. The experiments were conducted under the same conditions by using a calomel reference electrode and a gold counter electrode to set up a three-electrode configuration. An acetonitrile solution containing 0.10 M tetrabutylammonium hexafluoro-phosphate was used as the electrolyte.

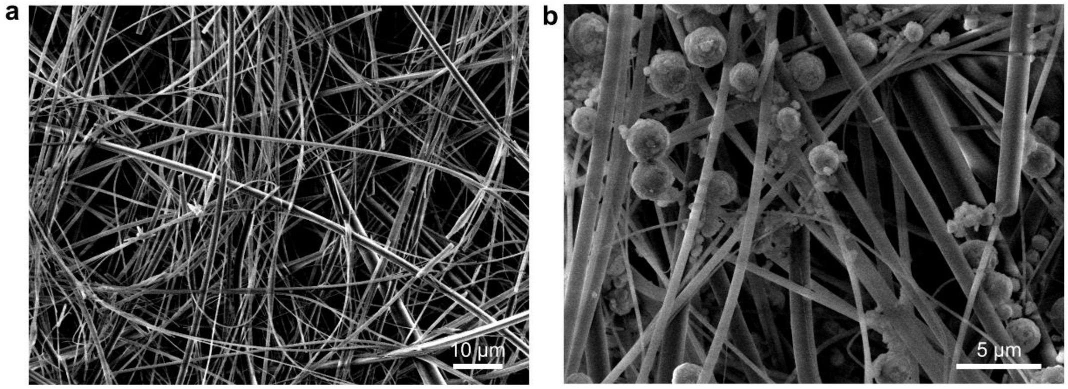

Supplementary Fig. 2 | SEM images of glass microfiber filter papers unloaded and loaded with catalytic particles. SEM images of glass microfiber filter papers (a) and the glass microfiber filter papers loaded with $SnIn_{0.1034}Cu_{0.0094}$ particles (b).

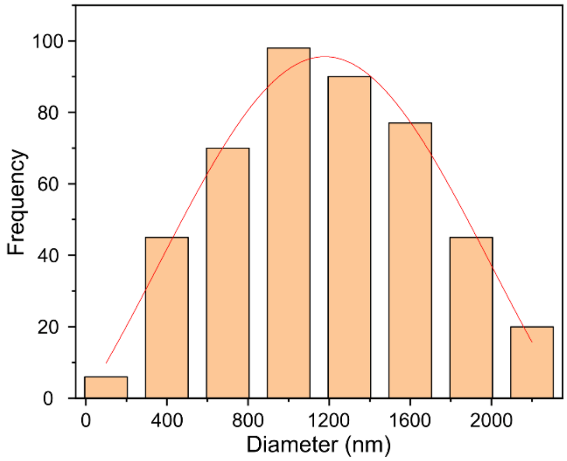

Supplementary Fig. 3 | Size distributions of $SnIn_{0.1034}Cu_{0.0094}$ particles. Size distributions of $SnIn_{0.1034}Cu_{0.0094}$ particles after the sonication process with the medium size of 1200 nm.

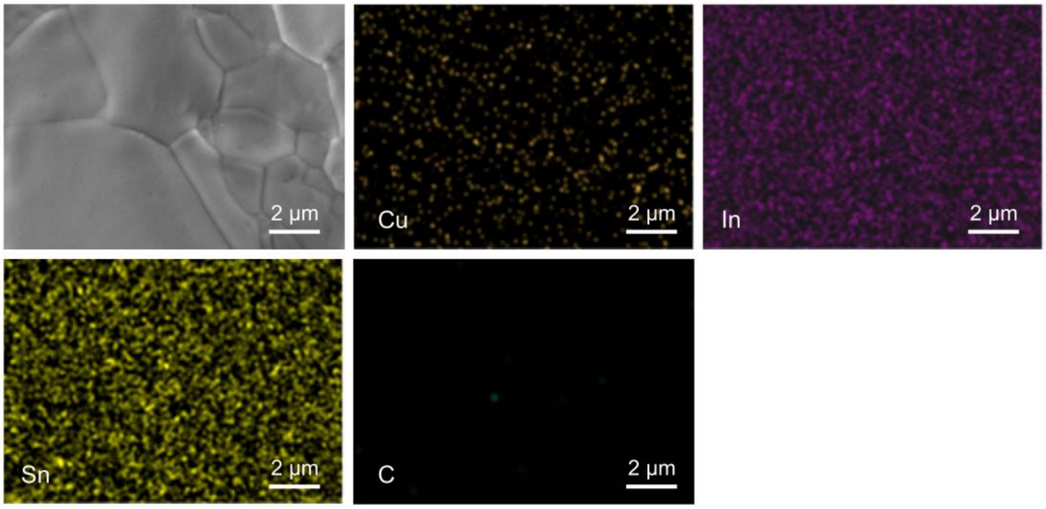

Supplementary Fig. 4 | SEM and the associated EDS images of $SnIn_{0.1034}Cu_{0.0094}$ after the reaction with hexadecane as feedstock. After the reaction, the alloy was gently washed several times with acetone to remove the residual liquid feedstock and then dried under vacuum. No solid byproducts were observed on the surface of the catalyst after the reaction.

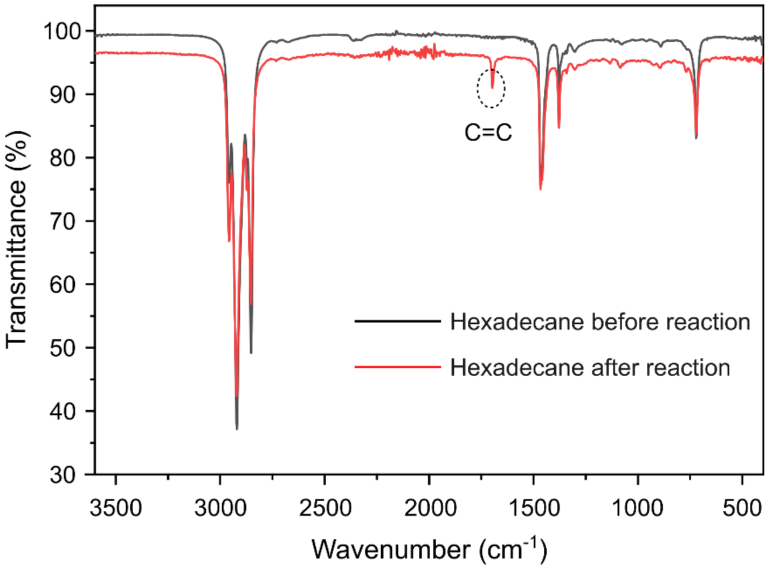

Supplementary Fig. 5 | FTIR spectra of hexadecane before and after reaction. The peak cantered at 1690 cm^{-1} after reaction represents the formation of C=C bonds, which indicates that C-H bond dissociation and H_2 formation were the dominant reactions.

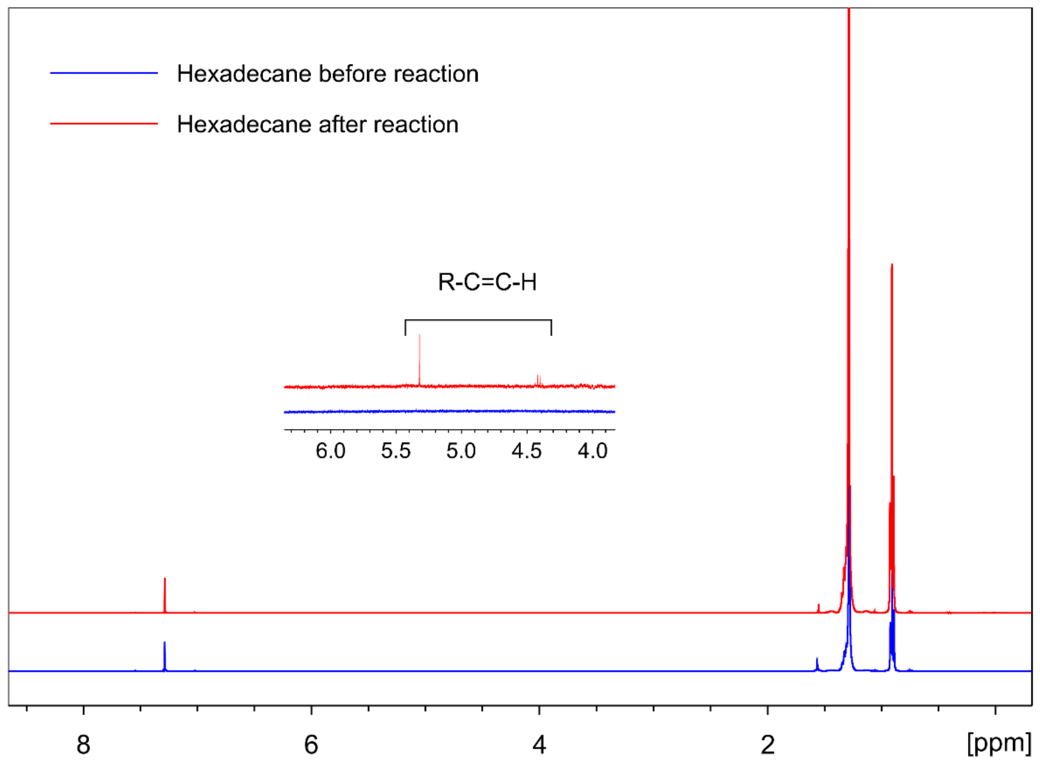

Supplementary Fig. 6 | NMR spectra of hexadecane before and after reaction. The new peaks can be assigned to the H bonded directly to a carbon-carbon double bond (-C=C-H). These results supported our proposed reaction mechanisms for H_2 generation from hexadecane. Hexadecane was dissolved into $CDCl_3$ for NMR analysis.

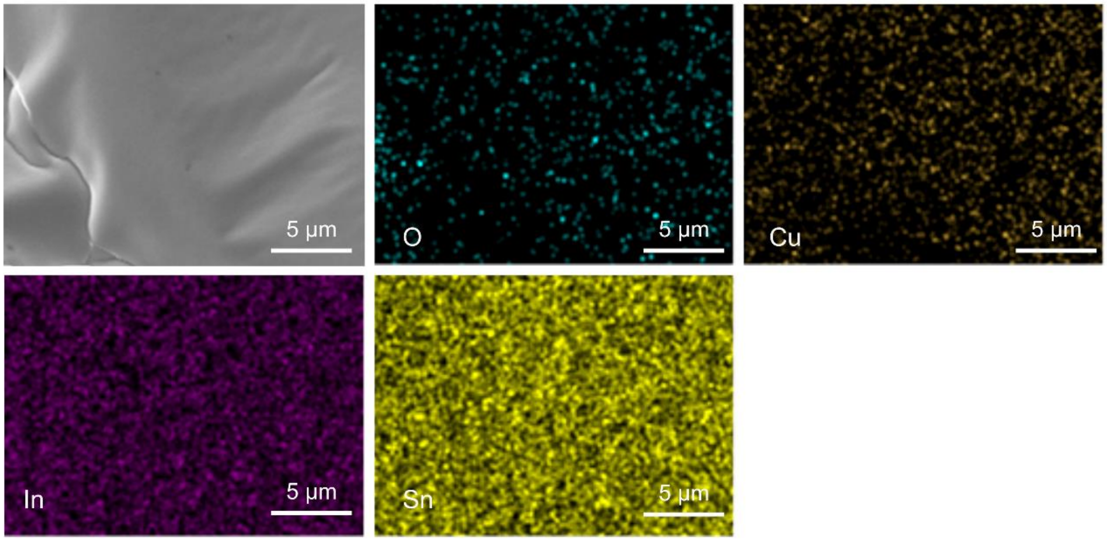

Supplementary Fig. 7 | SEM and the corresponding EDS images of the oxidized $SnIn_{0.1034}Cu_{0.0094}$ droplet. The sample was prepared by heating the $SnIn_{0.1034}Cu_{0.0094}$ droplet at ~260 °C under ambient conditions for several hours until the surface was completely oxidized.

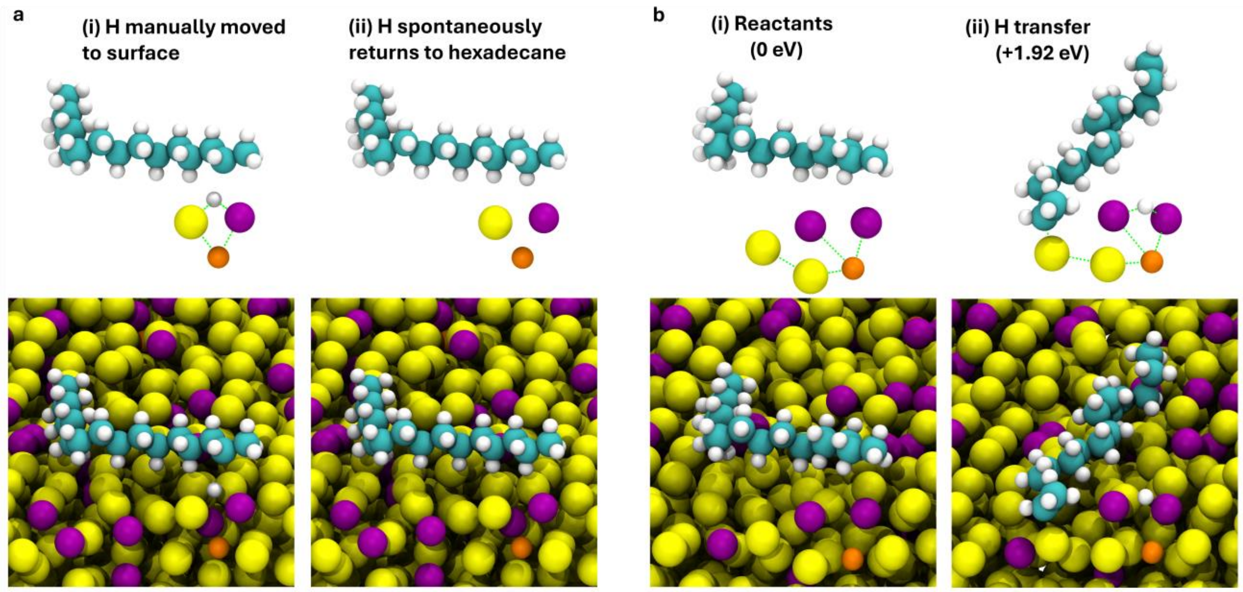

Supplementary Fig. 8 | Simulations for alternative atomic structures and the associated reaction pathways. a, Exemplar configuration where manual transfer of H from C-2 resulted in the spontaneous return of H to hexadecane in geometry optimization and MD simulation. **b,** Exemplar configuration where H remains on the surface, but the relative energy compared to the reactants is +1.92 eV.

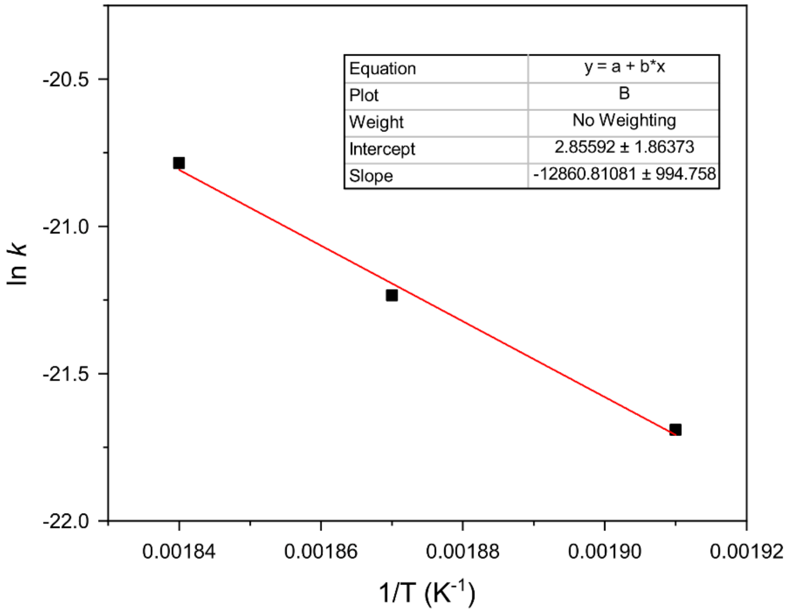

Supplementary Fig. 9 | Arrhenius plot for the calculation of the activation energies. The slope of the plot is calculated to be -12860 K in this case.

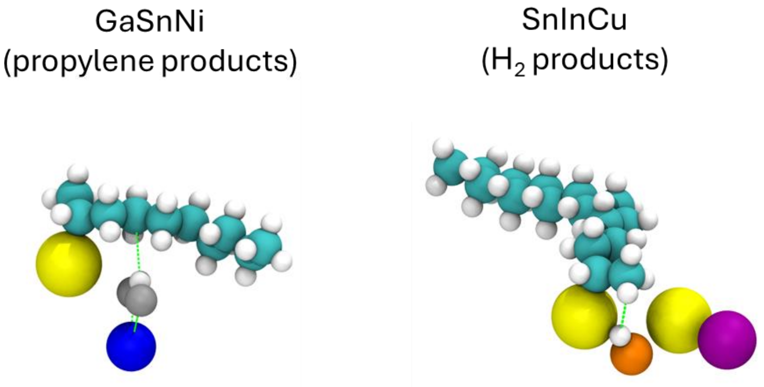

Supplementary Fig. 10 | Comparison of atomic structures within different metallic solvents. Comparison of the intermediate configuration of metaliic atoms for H remove from C-2 by using $GaSn_{0.029}Ni_{0.023}$ and $SnIn_{0.1034}Cu_{0.0094}$ as the catalysts, respectively.

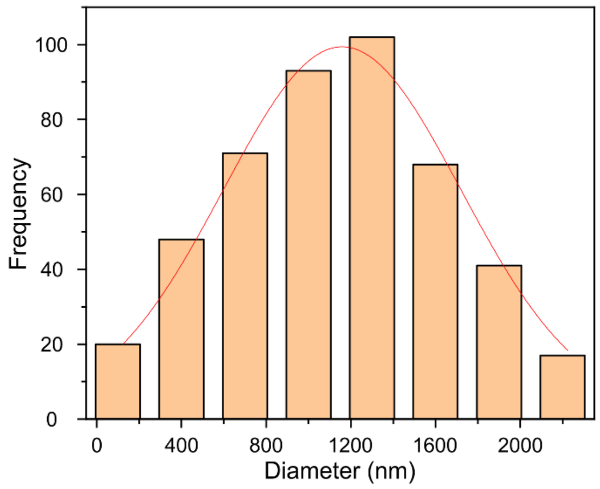

Supplementary Fig. 11 | Size distributions of catalytic particles. Size distributions of $SnIn_{0.1034}Cu_{0.0094}$ particles after reaction.

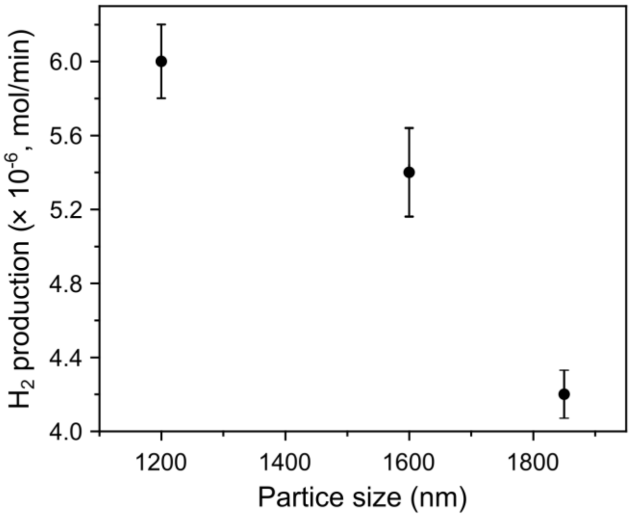

Supplementary Fig. 12 | H_2 production using $SnIn_{0.1034}Cu_{0.0094}$ particles of different sizes as the catalysts. The exposed surface area of $SnIn_{0.1034}Cu_{0.0094}$ particles increase as the particle size decreases. The particles were prepared by using a probe sonicator (model VC 750 from Sonics & Materials) under the protection of N_2 while being heated to ~300 °C to keep the alloy molten. The sonication amplitude was set to 55%, corresponding to an ultrasonic power input of ~410 W. The sonicator was set to pause for 1 s after each 9 s sonication. The sonication time was set for 10, 20 and 30 min to obtain $SnIn_{0.1034}Cu_{0.0094}$ particles with different sizes. Data are presented as mean values +/- SD, and n = 2 in each group.

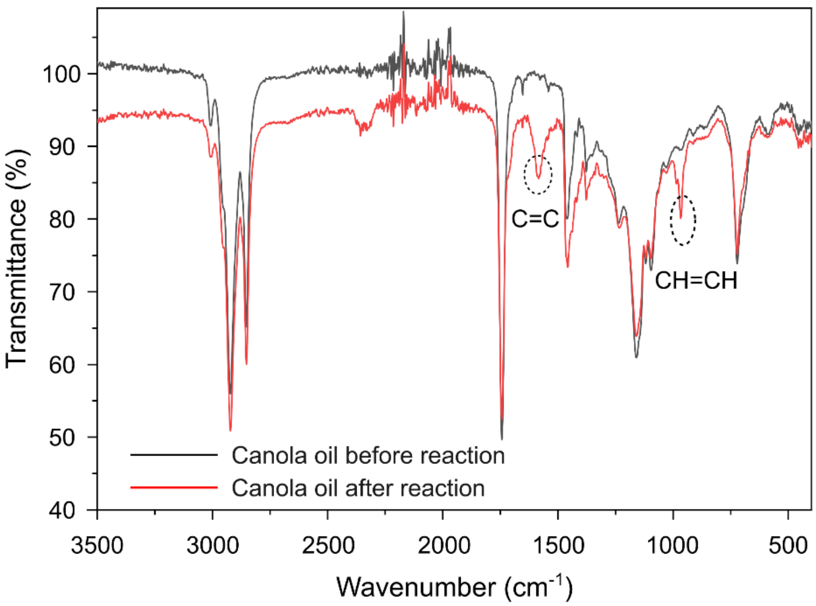

Supplementary Fig. 13 | FTIR analysis of canola oil before and after reaction. The peak centred at 1590 cm^{-1} after reaction is attributed to the C=C bond stretch, and the peak at 970 cm^{-1} corresponds to the formation of CH=CH within the long chain hydrocarbon. These results reveal that C-H bond dissociations and H_2 formations were the primary reactions during the conversion process of canola oil, indicating that canola oil likely went through the same reaction pathways as that of hexadecane.

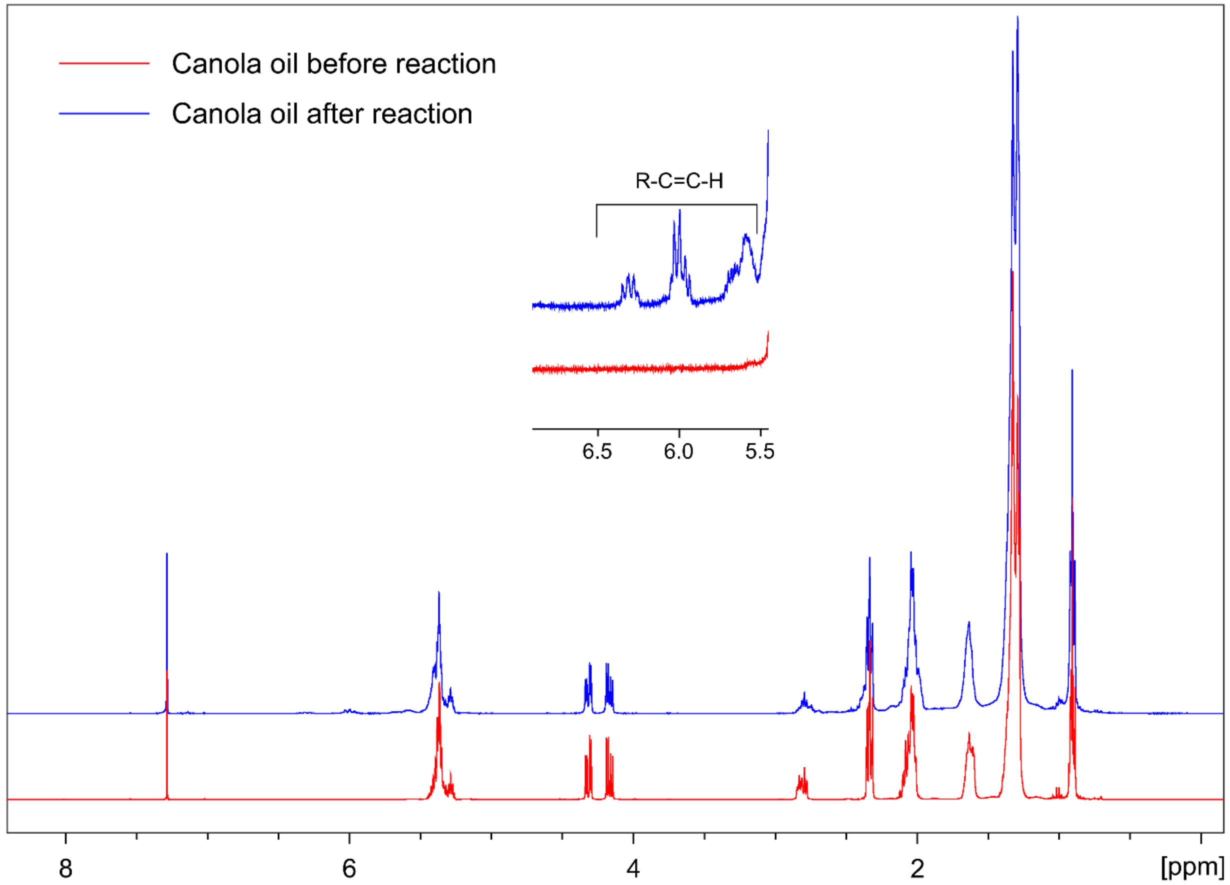

Supplementary Fig. 14 | NMR analysis of canola oil before and after reaction. The new peaks observed in the range of 5.5 to 6.5 ppm are attributed to H directly bonded to a C=C bond. These findings support the proposed mechanisms for hydrogen generation from canola oil. Conola oil was dissolved into $CDCl_3$ for further NMR analysis.

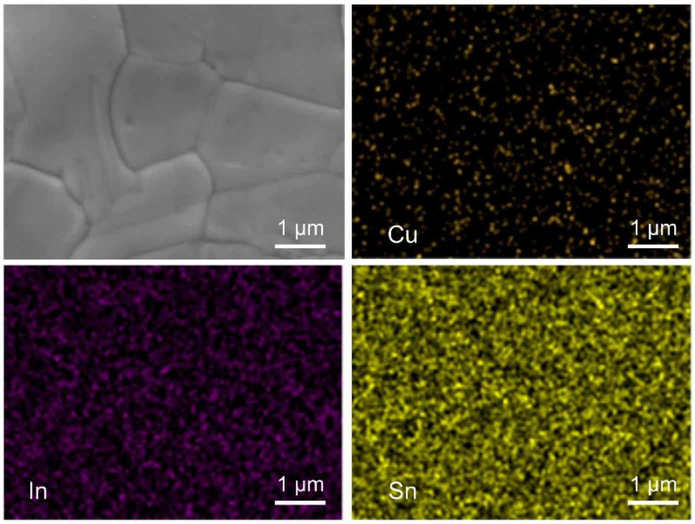

Supplementary Fig. 15 | SEM and EDS images of $SnIn_{0.1034}Cu_{0.0094}$ after the reaction with canola oil as feedstock. The alloy was gently washed by acetone several times and then dried under vacuum for further analysis.

Supplementary Table 1 | H_2 synthesis from hexadecane using different materials as catalysts

	Materials	Reaction temperature	Hydrocarbon source	Volume	Reaction time	H_2 (mol/min)	Ratio of H_2
Particles on glass microfiber filter papers		260 °C	hexadecane	15 ml	60 min	B.D.L.[(1)]	n/a
	$SnIn_{0.5169}Cu_{0.0094}$ 0.2g	260 °C	hexadecane	15 ml	60 min	5.8×10^{-7}	~98.5%
	$SnIn_{0.1034}Cu_{0.0094}$ 0.2g	260 °C	hexadecane	15 ml	60 min	6.0×10^{-7}	~98.0%
	$SnIn_{0.0518}Cu_{0.0094}$ 0.2g	260 °C	hexadecane	15 ml	60 min	1.4×10^{-7}	~96.5%
	$SnIn_{0.0103}Cu_{0.0094}$ 0.2g	260 °C	hexadecane	15 ml	60 min	8.0×10^{-8}	~89.0%

Note:
(1) B.D.L. represents beyond detection limits.
(2) The amount of other gaseous byproducts, including CH_4, C_2H_4 and C_3H_6, are not significant compared to H_2 and are not shown in the table.

Supplementary Table 2 | The influence of reaction temperature for H_2 synthesis

	Materials	Reaction temperature	Hydrocarbon source	Volume	Reaction time	H_2 (mol/min)	Ratio of H_2
Bulk materials	$SnIn_{0.1034}Cu_{0.0094}$ 5g	150 ºC	hexadecane	15 ml	60 min	B.D.L.[(1)]	n/a
	$SnIn_{0.1034}Cu_{0.0094}$ 5g	200 ºC	hexadecane	15 ml	60 min	B.D.L.	n/a
	$SnIn_{0.1034}Cu_{0.0094}$ 5g	260 ºC	hexadecane	60 min	60 min	3.6×10^{-8}	~98.0%

Note:
(1) B.D.L. represents beyond detection limits.

Supplementary Table 3 | H_2 synthesis using catalysts containing different amount of Cu

	Materials	Reaction temperature	Hydrocarbon source	Volume	Reaction time	H_2 (mol/min)	Ratio of H_2
Bulk materials (5 g)	$SnIn_{0.1034}Cu_{0.0009}$	260 ºC	hexadecane	15 ml	60 min	B.D.L.[(1)]	n/a
	$SnIn_{0.1034}Cu_{0.0018}$	260 ºC	hexadecane	15 ml	60 min	B.D.L.	n/a
	$SnIn_{0.1034}Cu_{0.0047}$	260 ºC	hexadecane	15 ml	60 min	7.2×10^{-9}	~85.0%
	$SnIn_{0.1034}Cu_{0.0094}$	260 ºC	hexadecane	15 ml	60 min	3.6×10^{-8}	~98.0%

Note:
(1) B.D.L. represents beyond detection limits.

Supplementary Table 4 | The influence of reaction temperature

Materials	Reaction temperature (K)	Hydrocarbon source	Volume	Reaction time	H_2 (mol/s)
$SnIn_{0.1034}Cu_{0.0094}$ (bulk 5g)	543	hexadecane	15 ml	60 min	9.4×10^{-10}
	533	hexadecane	15 ml	60 min	6.0×10^{-10}
	523	hexadecane	15 ml	60 min	3.8×10^{-10}

Supplementary Table 5 | H_2 production from canola oil using different materials as catalysts

	Materials	Reaction temperature	Hydrocarbon source	Volume	Reaction time	H_2 (mol/min)	Ratio of H_2
Particles on glass microfiber filter papers		260 °C	Canola oil	15 ml	60 min	B.D.L.[(1)]	n/a
	$SnIn_{0.5169}Cu_{0.0094}$ 0.2g	260 °C	Canola oil	15 ml	60 min	6.2×10^{-6}	~92.8%
	$SnIn_{0.1034}Cu_{0.0094}$ 0.2g	260 °C	Canola oil	15 ml	60 min	6.0×10^{-6}	~93.0%
	$SnIn_{0.0518}Cu_{0.0094}$ 0.2g	260 °C	Canola oil	15 ml	60 min	1.9×10^{-6}	~84.0%
	$SnIn_{0.0103}Cu_{0.0094}$ 0.2g	260 °C	Canola oil	15 ml	60 min	8.0×10^{-7}	~70.5%

Note:
(1) B.D.L. represents beyond detection limits.
(2) Other gaseous byproducts, including CH_4, CO, CO_2, C_2H_4 and C_3H_6, are not shown in the table owing to their insignificant quantity.

Supplementary Table 6 | Long-term experiment for H_2 generation from canola oil

Materials	Reaction temperature	Hydrocarbon source	Volume	Reaction time (h)	H_2 (mol/min)	Ratio of H_2
$SnIn_{0.1034}Cu_{0.0094}$ particles 0.2g	260 ^oC	Canola oil	15 ml	1	6.0×10^{-6}	~93.0%
	260 ^oC	Canola oil	15 ml	10	5.8×10^{-6}	~93.0%
	260 ^oC	Canola oil	15 ml	24	6.2×10^{-6}	~93.0%
	260 ^oC	Canola oil	15 ml	32	5.7×10^{-6}	~93.0%
	260 ^oC	Canola oil	15 ml	48	6.1×10^{-6}	~93.0%

Note:
(1) Other gaseous byproducts are not shown in the table owing to their insignificant quantity.

Supplementary Table 7 | H_2 synthesis using catalysts of different sizes

	Sonication time (min)	Medium diameter (nm)[1]	Reaction temperature	Hydrocarbon source	H_2 (mol/min)	Ratio of H_2
$SnIn_{0.1034}Cu_{0.0094}$ particles on glass microfiber filter papers (0.2 g)	30	1200	260 °C	Canola oil	6.0×10^{-6}	~93.0%
	20	1600	260 °C	Canola oil	5.4×10^{-6}	~93.0%
	10	1850	260 °C	Canola oil	4.2×10^{-6}	~93.0%

Note: (1) The particles were prepared by using a probe sonicator (model VC 750 from Sonics & Materials) under the protection of N_2 while being heated to ~300 °C to keep the alloy molten. The sonication amplitude was set to 55%, corresponding to an ultrasonic power input of ~410 W. The sonicator was set to pause for 1 s after each 9 s sonication. The sonication time was set for 10, 20 and 30 min to obtain $SnIn_{0.1034}Cu_{0.0094}$ particles with different sizes.

Supplementary Table 8 | Scaled-up experiments for H_2 production

Materials	Loading amount (g)	Reaction temperature	Hydrocarbon source	Volume	Reaction time (h)	H_2 (mol/min)	Ratio of H_2
$SnIn_{0.1034}Cu_{0.0094}$ particles on glass microfiber filter papers	0.2	260 ºC	Canola oil	50 ml	1	6.0×10^{-6}	~93.0%
	0.4	260 ºC	Canola oil	50 ml	1	1.1×10^{-5}	~93.0%
	0.6	260 ºC	Canola oil	50 ml	1	1.9×10^{-5}	~93.0%
	0.8	260 ºC	Canola oil	50 ml	1	2.3×10^{-5}	~93.0%
	5.0	260 ºC	Canola oil	200 ml	1	1.2×10^{-4}	~93.0%

Supplementary Table 9 | The influence of hydrocarbon feedstocks for H_2 synthesis

	Materials	Reaction temperature	Hydrocarbon source	Volume	Reaction time	H_2 (mol/min)	Ratio of H_2
Particles loaded on glass microfiber filter papers	$SnIn_{0.1034}Cu_{0.0094}$ 0.2g	260 ºC	hexadecane	15 ml	60 min	6.0×10^{-7}	~98.0%
	$SnIn_{0.1034}Cu_{0.0094}$ 0.2g	260 ºC	oleic acid	15 ml	60 min	1.8×10^{-5}	~84.5%
	$SnIn_{0.1034}Cu_{0.0094}$ 0.2g	260 ºC	canola oil	15 ml	60 min	6.0×10^{-6}	~93.0%

Supplementary Table 10 | Comparison with reported technologies for dehydrogenation reactions

Catalytic materials	Feedstock	Reaction temperature (ºC)	Pressure	Reaction types	Selectivity (%)	Efficiency ($mol \cdot min^{-1} \cdot g_{catalyst}^{-1}$)$^{(1)}$	Ref.
Pt–Sn/CeO_2	Propane	680	n/a	Dehydrogenation reaction	84.5	~8.0×10^{-2}	2
[$PtZn_4$]	Propane	520-620	n/a	Dehydrogenation reaction	< 95.0	~1.3	3
PtGa-Pb	Propane	600	n/a	Dehydrogenation reaction	99.6	~8.0×10^{-3}	4
Pt-Sn/Mg-Al	Methylcyclohexane	300	n/a	Dehydrogenation reaction	~90.5	~5.0×10^{-3}	5
Pt/TiO_2	Methylcyclohexane	400	n/a	Dehydrogenation reaction	99.9	~2.0×10^{-2}	6
$Ga_{52}Pt/SiO_2$	Methylcyclohexane	450	1 bar	Dehydrogenation reaction	85.0	~7.2×10^{-5}	7
Ga-Pd	Butane	445-500	1.1 bar	Dehydrogenation reaction	85.0	n/a	8
Ga−Rh	Propane	550	n/a	Dehydrogenation reaction	92.0	~5.8×10^{-3}	9
$Ga_{84}Pt/Al_2O_3$	n-heptane	410-470	n/a	Dehydrogenation reaction	~80.0	~4.5×10^{-4}	10
$SnIn_{0.1034}Cu_{0.0094}$	Canola oil	260	n/a	Dehydrogenation reaction	~93.0	~2.4×10$^{-5\ (2)}$	This work

Notes: (1) The efficiencies of the catalysts were calculated based on the weight of functional materials without including the weight of supporting species. (2) The rate was calculated based on the scald-up experiment by using 5.0 g of $SnIn_{0.1034}Cu_{0.0094}$ droplets (not just the surface) as catalytic materials. Considering only the surface area, the estimated production rate is ~9.0×10^{-3} $mol \cdot min^{-1} \cdot g_{catalyst}^{-1}$.

Supplementary References

1. Tang J., Christofferson, A. J., Sun, J., Zhai, Q., Kumar, P. V., Yuwono, J. A., Tajik, M., Meftahi, N., Tang, J., Dai, L., Mao, G., Russo, S. P., Kaner, R. B., Rahim, M. A., Kalantar-Zadeh, K. Dynamic configurations of metallic atoms in the liquid state for selective propylene synthesis. *Nat. Nanotechnol.* **19**, 306-310 (2024).
2. Xiong H., Lin, S., Goetze, J., Pletcher, P., Guo, H., Kovarik, L., Artyushkova, K., Weckhuysen, B. M., Datye, A. K. Thermally stable and regenerable platinum–tin clusters for propane dehydrogenation prepared by atom trapping on ceria. *Angew. Chem. Int. Ed.* **56**, 8986-8991 (2017).
3. Chen S., Zhao, Z.-J., Mu, R., Chang, X., Luo, J., Purdy, S. C., Kropf, A. J., Sun, G., Pei, C., Miller, J. T., Zhou, X., Vovk, E., Yang, Y., Gong, J. Propane dehydrogenation on single-site [$PtZn_4$] intermetallic catalysts. *Chem*. **7**, 387-405 (2021).
4. Nakaya Y., Hirayama, J., Yamazoe, S., Shimizu, K.-i., Furukawa, S. Single-atom Pt in intermetallics as an ultrastable and selective catalyst for propane dehydrogenation. *Nat. Commun.* **11**, 2838 (2020).
5. Yan J., Wang, W., Miao, L., Wu, K., Chen, G., Huang, Y., Yang, Y. Dehydrogenation of methylcyclohexane over PtSn supported on MgAl mixed metal oxides derived from layered double hydroxides. *Int. J. Hydrog. Energy*. **43**, 9343-9352 (2018).
6. Yang X., Song, Y., Cao, T., Wang, L., Song, H., Lin, W. The double tuning effect of TiO_2 on Pt catalyzed dehydrogenation of methylcyclohexane. *Mol. Catal.* **492**, 110971 (2020).
7. Sebastian O., Nair, S., Taccardi, N., Wolf, M., Søgaard, A., Haumann, M., Wasserscheid, P. Stable and selective dehydrogenation of methylcyclohexane using supported catalytically active liquid metal solutions – $Ga_{52}Pt/SiO_2$ SCALMS. *ChemCatChem*. **12**, 4533-4537 (2020).
8. Taccardi N., Grabau, M., Debuschewitz, J., Distaso, M., Brandl, M., Hock, R., Maier, F., Papp, C., Erhard, J., Neiss, C., Peukert, W., Görling, A., Steinrück, H. P., Wasserscheid, P. Gallium-rich Pd–Ga phases as supported liquid metal catalysts. *Nat. Chem.* **9**, 862-867 (2017).
9. Raman N., Maisel, S., Grabau, M., Taccardi, N., Debuschewitz, J., Wolf, M., Wittkämper, H., Bauer, T., Wu, M., Haumann, M., Papp, C., Görling, A., Spiecker, E., Libuda, J., Steinrück, H.-P., Wasserscheid, P. Highly effective propane dehydrogenation using Ga–Rh supported catalytically active liquid metal solutions. *ACS Catal.* **9**, 9499-9507 (2019).
10. Sebastian O., Al-Shaibani, A., Taccardi, N., Haumann, M., Wasserscheid, P. Kinetics of dehydrogenation of n-heptane over GaPt supported catalytically active liquid metal solutions (SCALMS). *React. Chem. Eng.* **9**, 1154-1163 (2024).
