# Supplementary material for: Molten Sn solvent expands liquid metal catalysis
Source: Nat Commun. 2025 Jan 21;16:907. doi: 10.1038/s41467-025-56222-0 (PMC11751482; doi:10.1038/s41467-025-56222-0)
Supplement: Supplementary file 2 — Description of Additional Supplementary Files [file 41467_2025_56222_MOESM2_ESM.pdf]

Description of Additional Supplementary Files

File Name: Supplementary Movie 1
Description: This movie shows the produced gaseous bubbles from the scale-up experiment using $SnIn_{0.1034}Cu_{0.0094}$ as the catalyst and canola oil as the feedstock. In the reactor, $SnIn_{0.1034}Cu_{0.0094}$ particles (0.8 g) were loaded on glass microfiber filter papers, which were stacked and immersed into canola oil. Then, the reaction proceeded with the reaction temperature maintained at ~260 °C. Gaseous bubbles were observed during the reaction. This movie was taken 2 hours after the start of the reaction. According to the gas chromatography measurement, the produced gas was mainly comprised of H_2 (~93.0% selectivity)."
